# Nickel-Based Catalysts for CO_2_ Methanation Supported on Plant Biomass-Based Activated Carbons—A Comprehensive Review

**DOI:** 10.3390/ma19112194

**Published:** 2026-05-22

**Authors:** Kamil Dudek, Piotr Żabiński

**Affiliations:** Faculty of Non-Ferrous Metals, AGH University of Science and Technology, Mickiewicz Avenue 30, 30-059 Cracow, Poland; zabinski@agh.edu.pl

**Keywords:** methanation, catalyst, fuel, biomass, nickel, activated carbon

## Abstract

The catalytic hydrogenation of carbon dioxide (CO_2_) to methane (CH_4_), commonly known as the Sabatier reaction, is a promising pathway for carbon capture and utilization (CCU). Nickel-based catalysts are cost-effective alternatives to noble metal systems, especially when supported on activated carbons derived from plant biomass. This review critically examines the basics of CO_2_ methanation, the role of catalyst composition and support materials, and the growing interest in biomass-derived activated carbons. Special emphasis is placed on synthesis routes, physicochemical properties, catalytic performance, and sustainability aspects. A comparative assessment of catalysts derived from different biomass sources is included, pointing out the most important factors influencing activity, durability, and economic feasibility.

## 1. Introduction to CO_2_ Methanation: Definition, Applications, and Significance

The upcoming energetic crisis is inevitable, and firm actions must be taken to avoid negative consequences connected with this event [1,2,3,4,5]. Moreover, international organizations, including the European Union and the United Nations, establish restrictions for CO_2_ emissions (Montreal and Kyoto protocols, Paris Agreement) [6,7,8]. Consequently, the modern industry has to modify its strategy to meet all the challenging laws and requirements and maintain productivity at a decent level. The answer to these challenges is a CO_2_ methanation reaction [9,10,11,12,13], which is an essential part of Power-to-Gas systems [14,15,16] that considers the conversion of surplus electricity from renewable sources (solar, wind, and water) to methane, a low-emission, clean fuel. There are already developed storage and transportation systems for methane, as it is the main component of natural gas. Thus, it is a convenient energy carrier. In addition, methanation makes it possible to utilize useless and abundant CO_2_. Hence, methane is strongly integrated into circular carbon economy frameworks. It is compatible with global decarbonization strategies, allowing the reduction of greenhouse gases in the atmosphere, and the utilization of the captured carbon dioxide at the same time. There is a possibility of combining CO_2_-capturing systems [17,18,19,20] with renewable hydrogen production facilities [21,22,23,24,25] and CO_2_ methanation reactors to create complete methane sourcing stations that are entirely based on renewable sources.

CO_2_ methanation relies on catalytic hydrogenation of carbon dioxide with hydrogen to produce methane and water:

$${\mathrm{CO}}_{2}+4H_{2}\to {\mathrm{CH}}_{4}+2H_{2}O\;\;\;\Delta H=-165\mathrm{kJ}/\mathrm{mol}$$

The most frequently proposed reaction mechanism is a two-step model that considers the formation of CO and water in the first stage, which is basically a reversed water gas shift:

$${CO}_{2}+H_{2}⟶CO+H_{2}O\;\;\;\;\;\;\;\;\;\;\;\;\;\;\;\;\Delta H=41\;kJ/mol,$$

which is an endothermic reaction. The second stage is exothermic and results in methane formation with water as a byproduct:

$$3H_{2}+CO⟶{CH}_{4}+H_{2}O\;\;\;\;\;\;\;\;\;\;\;\;\;\Delta H=-206\;kJ/mol$$

This highly exothermic reaction is thermodynamically favored at low temperatures and high pressures, although kinetic limitations necessitate the use of efficient catalysts [26]. The exothermic nature of this reaction demands very precise heat management. According to the literature [27], the optimal operating temperature is relatively low, ranging from 200 to 400 °C, resulting in nearly 100% methane selectivity, but raising the temperature up to 500 °C increases the reaction rate. Above this limit, a reversed water gas shift is likely to occur, and the methane yield drops rapidly. Lower methanation temperatures are also preferred because of catalyst deactivation risk due to carbon deposition at the material’s surface, but the detrimental influence of temperature increasing can be mitigated at a certain point by increasing the reaction pressure. For example, the experimental results have shown that increasing the pressure 11 times moves the carbon deposition temperature up by 40% [28]. Moreover, at higher pressures, a better methane selectivity is observed. Therefore, although the key factor deciding about methane yield is the reaction temperature, careful pressure adjustments can greatly contribute to optimization of the catalyst’s working conditions.

Gas hourly space velocity (GHSV) is a ratio between the volumetric flow of the reactants at 25 °C and 1 MPa pressure, and the total volume of used catalyst. It is another parameter determining catalysis’ effectiveness. As shown in the study conducted with the use of Ni-based catalyst supported on mixed metal oxides [29] at the temperatures exceeding 350 °C, changing GHSV does not affect methane yield significantly. However, below this temperature, higher CH_4_ yields are observed at lower GHSV, as the reaction runs under thermodynamic equilibrium. The CO selectivity is also dependent on GHSV and rises at higher rates, as CO molecules are moving faster and are less likely to be hydrogenated.

The reagents’ ratio is a parameter that affects both catalytic performance and the economy of CO_2_ methanation. Minimization of H_2_ amount in the feed mixture reduces expenses, as supplying the methanation system with this gas generates the main costs. On the other hand, increasing the H_2_/CO_2_ ratio improves both CH_4_ yield and selectivity. Furthermore, a low H_2_/CO_2_ ratio means high risk of carbon deposit formation—for instance, if the ratio is 2, a broad coke deposition is observed under 500 °C [30]. In contrast, if the hydrogen to carbon dioxide ratio is 4 or above, coke deposition is minimal and does not affect the catalyst performance significantly. Decreasing the ratio below 4 causes unavoidable carbon deposition, even when increasing the reaction pressure up to 30 MPa. A proper management of Gibbs free energy allows even total CH_4_ selectivity under 500 °C, when the H_2_/CO_2_ ratio is held at 4 [31].

## 2. Catalysts for CO_2_ Methanation

Catalysts for this particular reaction are mostly based on highly dispersed particles of transition metals. Ru and Ni are especially valuable, as they give exceptionally high CH_4_ selectivity [32].

**Figure 1:**
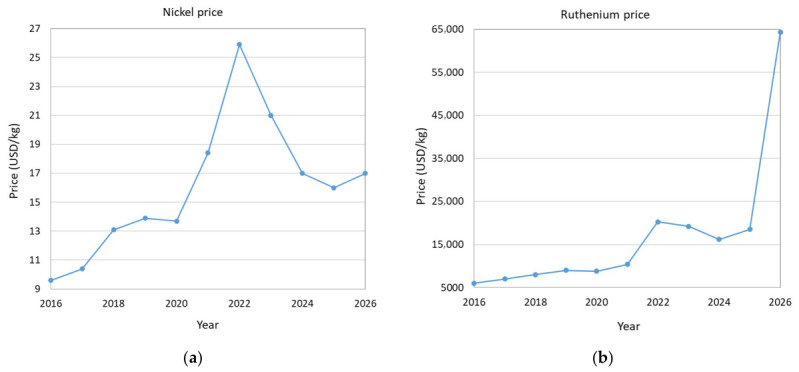
Average Ni (**a**) [33,34] and Ru (**b**) [35] prices from 2016 to 2026.

The prices of Ni and Ru in the years 2016–2026 are presented in Figure 1. For Ni (a), average annual global prices were considered. A significant Ni value growth occurred up to 2022—it stems from supply shock and a high demand for resources from the battery sector. It was also connected to the start of the Russo-Ukrainian war, when the main supply chains were broken. Afterwards, a correction and stabilization were observed. In the case of Ru (b), data from the 2020–2026 period and careful estimates for 2016–2019 were used, as the Ru market is largely opaque and its historical data are limited. The Ru prices were relatively stable until 2020 (COVID pandemic), then the rapid growth was observed, and in 2026, a giant leap occurred as a consequence of technological demand connected to the Russo-Ukrainian war.

In the case of Re, Mo, Rh, Pt, Au, and Pd-based catalysts, significant amounts of CH_3_OH and CO are also observed in post-reaction mixtures. Furthermore, Cu and Ag-based catalysts offer CH_3_OH as the main CO_2_ methanation product [36].

Transition elements are divided into noble and common metals. Despite their very good performance and durability, noble metals are rarely used for large-scale synthesis due to high precursor prices and limited availability, making them unsuitable for industrial-scale production. Common metals, such as Ni, Co, and Fe, are relatively cheap and abundant. These features make a strong potential for their large-scale, industrial application in CO_2_ methanation. Due to its high activity and low cost, nickel is the most widely used element in this group [37], but its disadvantage is a high sensitivity to sulfur presence in the starting gases mixture, so it requires reactants’ desulfurization to avoid quick catalyst deactivation [38].

CO_2_ methanation catalysts can be supported on various materials, and among them, metal oxides (e.g., Al_2_O_3_, SiO_2_, TiO_2_, CeO_2_, ZrO_2_) are the most commonly applied group. Al_2_O_3_ and SiO_2_ are particularly interesting regarding their strong early performance. Unfortunately, these materials are prone to sintering and carbon deposition at elevated temperatures [39], yet the Al_2_O_3_-supported systems are widely distributed for commercial use (e.g., by BASF, Evonik, Clariant, Topsoe, and Johnson Matthey). Composite supports for Ni-based CO_2_ methanation catalysts are gaining strong attention as well, as they significantly improve catalytic activity. These systems are based on at least two commonly used oxides. For example, Ni supported on ZrO_2_-Al_2_O_3_ composite exhibits better dispersion and higher reducibility, as the ZrO_2_ blocks the immersion of Ni species into the lattice of γ-Al_2_O_3_. These interactions result in higher CO_2_ conversion and improved CH_4_ selectivity [40]. Mixed oxide supports and composites with carbon materials create synergistic effects that improve catalytic performance [41,42]. Components like TiO_2_ or CeO_2_ can introduce electronic effects, such as Ti^3+^ ions and oxygen vacancies, enhancing metal dispersion and reducibility [43,44]. Moreover, it is possible to choose a preferred CO_2_ methanation route by changing the support composition, as the reaction typically follows three pathways: the CO pathway, the RWGS + CO-hydro pathway, or the formate pathway [45,46]. This opens the possibility for further catalyst customization and adjustments. The quality of support can be further improved by incorporating promoters in catalytic systems. Fe works as the H_2_ adsorber, as the polycrystalline iron shows a higher affinity to hydrogen compared to Ni, thereby improving the catalyst’s performance [47]. Alkaline earth metals, such as Mg, can stabilize the structure of catalyst support, as shown in a study by Shen et al. [48]. Mg addition eliminated acid sites from the obtained material’s surface and enhanced rates of CO_2_ activation, improving methane yield. The La addition increases Ni reducibility, as it compromises bonds between nickel and the matrix. Additionally, this element creates medium-strength basic sites, improving carbon dioxide adsorption rates. This results in obtaining higher CH_4_ yields at lower temperatures [49]. All in all, increasing support’s basicity helps to form a monodentate species of formate, significantly improving the catalyst’s selectivity [50,51]. Cerium accelerates Ni reduction by incorporating Ni^2+^ into the ceria lattice, which results in the replacement of Ce^4+^ ions and nickel-ceria solid solution formation [52]. Consequently, saturated oxygen vacancies are generated. Thus, the highly reactive oxygenated species are formed, which can be easily reduced even at low temperatures [53]. Magnesium introduces weak or moderate (depending on support) basic sites to the catalyst’s surface. These sites enhance the adsorption of carbon dioxide as carbonate or bicarbonate species, which are subsequently hydrogenated to methane through intermediate species such as formates [54,55,56]. Manganese improves catalyst reducibility, enhances CO_2_ adsorption, and increases the surface area, thereby improving catalytic performance and selectivity. Mn-doped catalysts often follow the intermediate route for CO_2_ methanation. Such a pathway considers the formation of Ni^0^ or Ni^2+^ species and oxygen vacancies, facilitating carbon dioxide methanation [57,58,59]. Nitrogen stabilizes key intermediates on carbonic supports. For example, pyridinic nitrogen in nitrogen-doped carbon nanotubes makes it easier to bind CO_2_ molecules and reduces the overpotential for carbon dioxide reduction, improving both selectivity and catalytic activity. Pyridinic nitrogen is particularly effective because its free electron pair can bind CO_2_ [60,61].

## 3. Activated Carbon

Activated carbon (AC) is a material with various applications. Its large specific surface area (above 1000 m^2^/g) and great adsorption capabilities make it useful in many industrial sectors, including petroleum, agriculture, pharmacy, cosmetics, automotive, textiles, and more [62]. Nowadays, a broader spectrum of AC applications is studied by researchers. ACs are considered to be used as energy storage system components [63,64,65,66,67,68], pollutant removal [69,70,71], CO_2_ capture systems [72], etc. Moreover, ACs’ tunable physicochemical properties allow using them as catalyst supports for the oxygen reduction reaction (ORR) [73,74,75,76,77], hydrogen evolution reaction (HER) [24,64,78,79], biofuel production [80,81,82], methane reforming [83], tetracycline degradation [84], methane production from syngas [85], furfural hydrogenation [86], lipid feedstocks hydroprocessing [87], lignin-type compounds conversion [88,89], lactic acid production [90], hydrogen peroxide sensing [91], steam reforming of pyrolytic oil [92], CO_2_ reduction [93], and more.

The first step of an activated carbon (AC) preparation is making a biochar, an initially carbonized organic material. The International Biochar Initiative defines biochar as “a solid material produced from the thermochemical conversion of biomass in an oxygen-limited environment.” [94]. Biochar is mainly made of solid biological waste or simply biomass. It is cheap and commonly accessible, being an abundant renewable energy source [95,96].

Pyrolysis is the most popular method of biochar production, relying on thermal decomposition of organic matter without access to oxygen at temperatures of 300–900 °C. Most of the biochar precursors consist of wood-like biomass, and its main components are cellulose, hemicellulose, and the hardest component to pyrolyze—lignin [97]. Pyrolysis consists of three stages [98,99,100]; the first one is the loss of free moisture and some percent of volatiles. In the second one (which occurs at 200–500 °C), the organic matter decomposes, including cellulose, hemicellulose, and a part of lignin—this is the primary pyrolysis. When the temperature still rises, lignin finally decomposes, closing the pyrolysis process. Pyrolysis can be fast or slow; the slow variant takes from a few minutes to several days, and the yield of biochar is typically 15–89%. Fast pyrolysis is conducted at a very high heating rate, typically from 10 to 1000 °C/min, and the residence time is very short, ranging from 0.5 to 2 s. Bio oil is the main product of such pyrolysis, and its maximum yield is reached at 500 °C [101,102], but the biochar yield is significantly reduced—it reaches 12–37% [103]. Fast pyrolysis also requires high raw material fragmentation (grains smaller than 3 mm in diameter) due to the weak biomass’s thermal conductivity and the necessity of rapid heat transfer. Alkali or alkaline earth metals can support pyrolysis by secondary cracking promotion of volatiles, resulting in additional gas product formation and enhancing the biochar formation [104,105].

Partial gasification is another method of biochar production, which relies on endothermic decomposition of organic matter into gaseous products and biochar, conducted in oxygen-containing gases, such as air, steam, or pure oxygen, at temperatures ranging from 700 to 1500 °C. Steam gasification is particularly interesting, as it allows obtaining renewable hydrogen along with high-quality biochar [106], but its yield is relatively low. On the other hand, such biochar contains large amounts of alkaline earth metals, e.g., Na, K, Mg, Ca, etc., as well as polycyclic aromatic hydrocarbons, especially beneficial for catalytic applications [107].

Hydrothermal carbonization is the thermochemical decomposition of biomass, performed at relatively low temperatures, ranging from 180 to 250 °C. It utilizes anoxic conditions and high pressure (14–22 MPa), along with subcritical water as a decomposing agent. Because of that, hydrothermal carbonization’s performance is not dependent on the water content of the raw material. During the process, gaseous (mostly CO_2_), liquid (bio oil + water), and solid (biochar) products are formed. In comparison with pyrolysis and partial gasification, hydrothermal carbonization is cheaper, as it does not require biomass drying. It can be conducted in milder conditions and generates fewer detrimental byproducts [108,109]. Unfortunately, using this method also means obtaining a less stable biochar, due to its lower aromatic compound content compared to biochars produced by other methods [110].

The quality of biochar is assessed by its physicochemical properties. Pore volume, pore size, and specific surface area (SSA) are the key physical parameters of biochars. During pyrolysis, the release of volatile compounds and dehydration causes porosity development [111], improving SSA and enhancing metal ion adsorption. Biochar catalyst support forces the flow of reactant gases through pore channels, increasing their diffusion and improving heat distribution, making the catalyst more resistant to sintering and poisoning [112]. The structure of biochar strongly depends on the type of biomass utilized as a carbon source. Biochar can be obtained from diverse sources, such as forestry residues [113,114,115,116], algae [117,118,119], agricultural wastes [120,121], or even animal manure [122]. Material derived from plant biomass exhibits higher porosity than animal-originated material, which is connected to lignocellulosic matter’s tubular structure, resulting in a larger number of micropores. The higher amount of micropores translates into larger SSA, while the total pore volume is determined by macro- and mesopores incorporation. Pyrolysis temperature strongly impacts the character of the resulting biochar. The material obtained at lower temperatures exhibits an amorphous carbon structure, but increasing decomposition temperature causes the loss of hydrogen- and oxygen-containing functional groups and improves graphitization. Up to a certain level (mostly 900 °C), there is a positive correlation between pyrolysis temperature and the level of porosity, along with the size of SSA. Increasing decomposition temperature above this value results in collapsing, blocking, or even fusion of the micropores, which reduces the SSA [123,124,125,126].

Chemical properties of biochar are equally important. The first parameter is the alkali or alkaline earth metals content. Incorporation of light metals like Na, K, Mg, or Ca improves support basicity and causes the formation of basic active centers, greatly contributing to CO_2_ activation during the methanation. Alkaline earth metals also strengthen the metal–support interaction, preventing active phase sintering [127], as well as induce additional oxygen deposits to the support’s surface, increasing its oxidizing properties, thus protecting the active phase from carbon deposition and deactivation [128,129]. The common problem with biochars is the presence of sulfur, phosphorus, and chlorine, occurring naturally in biomass. P and S form strong bonds with active centers, blocking their access to reagents and excluding them from catalytic reaction [130,131]. The sulfur is particularly detrimental, as only a few ppm of this element is enough to cause a significant drop in catalytic activity [132]. Chlorine is less dangerous, because its adsorption to Ni is reversible [133], yet it still decreases the catalytic performance. To ensure long-term operation of a catalyst supported on AC, an additional purification step should be considered to exclude the possibility of catalyst poisoning. Removing poisons (e.g., by strong acid or base treatment) can significantly improve the catalyst’s activity and stability [134].

Biochar’s surface is covered with various oxygen-containing functional groups, including lactone, phenolic hydroxyl, carbonyl, hydroxyl, or carboxyl [135,136,137]. The groups that provide good adsorption capabilities are lactone, carbonyl, carboxyl, and phenolic hydroxyl. These functionalities also allow adjustment of surface charge, pH, and hydrophobicity or basicity [138,139,140,141,142,143,144]. Moreover, a large number of these functionalities affect the support’s hydrophilicity, thus improving its affinity towards metal ions, enhancing the impregnation efficiency [145]. In addition, their displacement determines active metal phase dispersion, as they act as the anchoring sites, chelating metal ions [146] by forming coordinating bonds between oxygen’s free electron pairs and empty d orbitals of transition metal atoms. Thus, the exposure of oxygen-containing functional groups (especially hydroxyl) strongly impacts the availability of anchoring sites [147]. It increases the complexation of metal ions, not only improving active phase dispersion, but also strengthening the metal–support interaction [148,149].

Consequently, properly tailoring the number of certain functional groups provides a potential to adjust the support to particular applications and, in the case of catalysts’ support, to specific conditions of catalyzed chemical reaction.

When the biochar is obtained, the next processing stage is activation. It can be conducted via physical or chemical methods. Activation is necessary to obtain a high-quality functional material, as the raw biochar generally exhibits poor porosity and modest SSA. These parameters are especially important if the carbon is meant to be used as a catalyst support, as the grade of this support will determine the nature of its interactions with the active phase.

Physical activation relies on heating the biochar in an oxidizing atmosphere, such as CO_2_, H_2_O, or O_2_. This type of activation requires high operating temperatures (500–1000 °C) [150]. It changes biochar’s porosity and its surface chemistry (polarity, functional groups, etc.). Another purpose of such activation is the further development of biochar’s SSA and pore structure [151]. The most popular activators are steam and carbon dioxide.

During the steam activation, oxygen from water molecules is attached to active sites on biochar’s surface, resulting in H_2_ evolution. This hydrogen forms complexes in reaction with surficial carbon atoms. Such a process causes SSA augmentation through the release of volatiles, which block the pores, and results in the implementation of additional functional groups along with surface modifications [150].

CO_2_ activation is easier to adjust due to the lower activity of this gas at high temperatures, and the tendency to micropore formation [152,153]. In contrast to steam activation, the CO_2_ variant favors microporosity development and SSA extension. It stems from comparable rates of CO_2_ diffusion speed through biochar structure and the reaction with its specific surface. Steam’s diffusion speed is lower than its reaction rate, so it does not penetrate biochar, but reacts only with its most accessible areas on the surface. Consequently, biochar activated with CO_2_ shows higher microporosity and larger SSA than its steam-activated counterpart [154].

Chemical activation considers the use of reagents, which decrease the activation temperature. It can be performed as a one-or two-step process. One-step chemical activation relies on raw material carbonization and its activation at the same time, while the two-step variant considers mixing the previously obtained biochar with chemicals to activate it [155]. The activators can be acids or alkali, and the most commonly used agents are KMnO_4_, ZnCl_2_, K_2_CO_3_, KOH, H_2_O_2_, or H_3_PO_4_ [156,157,158,159,160,161,162]. Most of these chemicals are caustic, so they are able to enhance porosity through carbon atom removal from the support, counteract the formation of tar, and cause the release of volatile compounds.

Acid activation is performed with the use of strong acids, such as HNO_3_ or H_3_PO_4_. It increases the number of surficial functional groups and changes pore structure. For example, phosphoric acid can degrade aromatic and aliphatic compounds, as well as lignocellulose, simultaneously creating phosphate and polyphosphate bridges. This process prevents constriction during pore spreading [163].

Alkali activation of biochar is related to the acid process, but it is more favorable towards SSA enlargement and pore structure development. Compared to acid activation, it is more frequently applied for biochar-based catalyst carriers synthesis, and the most commonly used alkaline activators are K_2_CO_3_, NaOH, and KOH [164]. The last one is labeled as the most efficient alkali activator of biochar, which is going to be applied as a catalytic support. This is due to its low activation temperature, large SSA, and beneficial micropores distribution of the activated carbon. Such material can adsorb a higher amount of well-dispersed active metals, providing an excellent contact between the catalyst’s active sites and reagents.

The optional modification of biochar is the heteroatom doping. It can be conducted via an in situ or post-treatment process [165]. The in situ option considers adding the dopant(s) during biochar preparation via high-temperature carbonization. Such heteroatoms, incorporated into the carbon lattice, can significantly increase charge delocalization, breaking the inertia of sp^2^-hybridized carbon structures and resulting in better catalytic performance. Post-treatment doping, implementing solid mixing and impregnation methods, can increase the number of heteroatomic functional groups on the surface of the catalyst, while not changing its other properties.

## 4. Biomass-Derived Activated Carbon-Supported Ni Catalysts

Although this specific group of catalysts for CO_2_ methanation is still a niche, it develops rapidly and is expected to become an important branch of a large family of CO_2_ reduction catalysts. Particular interest is observed in catalysts supported on waste materials, especially from the agricultural and timber industries.

**Figure 2:**
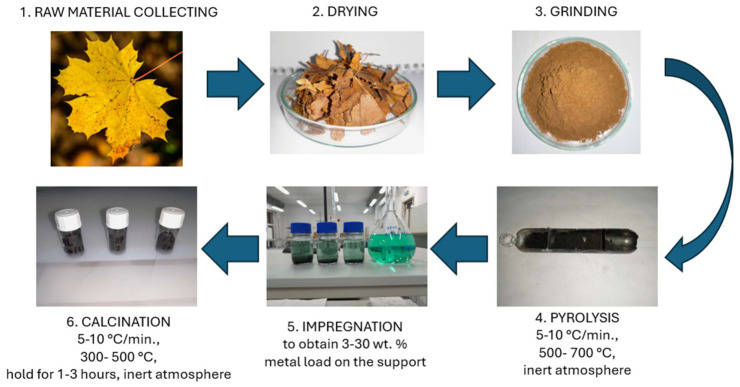
A scheme of a typical synthetic path for a metal-based catalyst supported on biomass-derived AC [170].

As shown in Figure 2, a standard synthetic procedure of biomass-derived AC-supported, metal-based catalyst consists of a few basic steps. However, numerous variations can occur, depending on the type of biomass used as a carbon source, the method of metal deposition, the incorporation of promoters, the type of carbon activation, etc. Utilizing AC as the catalyst support has a very strong asset. Such a catalyst can be recycled according to a very simple end-of-life strategy. The composite, which has undergone structural degradation and cannot be activated for use as a catalyst, can be burned as a fuel. The ashes remaining after composite combustion will still contain remains of an active phase. Next, these ashes can be treated with strong acids to convert the oxides of Ni, Ru, and their dopants (Ce, Ca, Mg, etc.) into ions, which can then be dissolved in water. Such a solution can be utilized for fresh support impregnation to obtain a catalyst again. In this way, the catalyst’s life cycle is closed, preventing waste generation [166,167]. In contrast, metal oxide-supported catalysts cannot be utilized as a fuel, and require more complex recycling procedures [168,169].

### 4.1. The Type of the Carbon Source

A particular interest is observed in agricultural waste biomass utilization. To begin with, Renda et al. [171] obtained a biochar from wheat straw through slow pyrolysis in a nitrogen atmosphere [172], and activated it with CO_2_ at 1 MPa, 700 °C [150]. This AC was further doped with CeO_2_ via wet impregnation (different concentrations of nickel nitrate hexahydrate were used to obtain 10, 30, and 50 wt. % Ce loads), and calcinated at 500 °C in an Ar atmosphere. Then, the doped biochar was analogically impregnated with nickel nitrate hexahydrate to deposit nickel on the support. The best-performing variant was the one with a 30 wt. % Ce load—it exhibited a 60% CH_4_ yield at 450 °C and 0.1 MPa. In the durability test conducted at the same operating conditions, the methane yield dropped from 60 to 30% after 14 h of constant methanation. The authors stated that the excessive Ce load causes pore plugging in the AC, which reduces the size of the specific surface area, consequently decreasing Ni dispersion. Since the catalyst with 50 wt. % The Ce load showed worse performance than the optimal variant with 30 wt. % Ce load. After aging tests, various sintering intensities were observed on different catalyst variants. The sintering intensity order was directly connected to the Ce load: 0 wt. % Ce > 10 wt. % Ce = 30 wt. % Ce > 50 wt. % Ce. This shows how important the Ce role is in active phase immobilization. It is crucial for sintering prevention and maintaining good catalyst performance.

A similar approach is visible in the study made by Di Stasi et al. [173], but aside from ceria, in this case, urea-doping is also considered. Wheat straw pellets were pyrolyzed at the same parameters as in [171]. Also, the experimental setup for pyrolysis was analogical as in their other study. Physical activation with CO_2_ of the obtained biochar was conducted under the same conditions as in [150], which appeared to be highly effective and provided a hierarchical pore size distribution in the resulting material. As above, the catalysts in this study were prepared with a wet impregnation method with cerium nitrate and urea as Ce and N precursors. Calcination was conducted at 550 °C for 3 h in a nitrogen atmosphere, and the wet impregnation was applied again for Ni implementation with nickel nitrate as the precursor of the active phase. Analogically to [171], different Ce and N loads were applied. The variant of the catalyst with 30 wt. % Ce and 20% wt. Ni showed the highest catalytic activity with 62% CH_4_ yield. The authors stated that this performance comes from the Ce doping effect by introducing oxygen vacancies, which have a significant affinity towards oxygen atoms in CO_2_ molecules [174]. Moreover, CeO_2_ addition causes the formation of basic sites, enhancing CO_2_ adsorption [175]. This dopant stabilizes and improves Ni dispersion as well, enhancing interaction between the active phase and the support. On the other hand, too high Ce load results in ceria shell formation, encapsulating the support and minimizing specific surface area, thereby reducing Ni dispersion [176] and decreasing the CO_2_ conversion level. Considering Ni load changes, decreasing it from 40 to 20 wt. % caused an improvement in CH_4_ selectivity from 25 to 100% at 350 °C, 1 MPa. Such a result is said by the authors to be connected to particle metal agglomeration [177], which means worse Ni dispersion across the support. N-doping increases support basicity, causing a beneficial effect of stronger CO_2_ adsorption [178].

Another agricultural waste biomass, which is a promising raw material for obtaining AC as a support for CO_2_ methanation catalysts, is rice husk. Chernyak et al. [134] developed a set of catalysts based on Ni, promoted with Mn, and supported on rice husk-derived AC. Their oxidized carbon-supported catalyst achieved 65% methane yield at 350 °C. In their work, an unusual approach to support synthesis was applied, as they developed a hybrid silica-carbon material by calcinating a rice husk-based biochar (obtained by pyrolysis at 600 °C) in nitrogen or air flow. In this way, it was possible to develop a unique SiO_2_/C-ox support structure. Ni and Mn were deposited on the support via the classic wet impregnation method, obtaining a Ni loading of 17 wt. %, and the Mn:Ni molar ratio of 1:10. Samples were annealed at 350 °C for 1.5 h in a nitrogen flow of 60 mL/min. A series of catalytic tests was conducted at 200–375 °C, 1 MPa for 4 to 8 h, with a volume H_2_:CO_2_ ratio of 4:1. Comparing the activity of synthesized materials, the authors have found that oxidizing the carbonaceous support hinders catalytic performance, which is connected to the presence of ultramicropores that interrupt reagents diffusion to and from the active sites. A beneficial effect on materials activity was observed after support purification with acids and bases in order to remove sulfur, phosphorus, alkali, and alkaline earth metals. Consequently, NaOH, HNO_3_, and HCl treatments lead to obtaining supports that allow achieving better CO_2_ conversion and methane selectivity.

Catalysts supported on AC obtained from whole plants or their parts are also widely studied. Yue et al. [179] decided to utilize both freshwater and seawater reed as the support precursors. The mowed straws were pyrolyzed at a 500–700 °C temperature range [180], with a 10 °C/min heating rate. It was proven that the higher the pyrolysis temperature, the better the stability of the resulting biochar [181], but on the other hand, the used calcination temperature should not exceed the temperature of the raw material’s pyrolysis due to the possibility of support damage. Thus, the obtained biochars were ground, calcinated at 500 °C for 3 h under N_2_ atmosphere, and reduced at a modest temperature of 300 °C, as the reduction temperature is not the main parameter determining the catalytic performance [182]. During pyrolysis, a significant amount of volatiles was released, including hydrogen that was directed to the catalyst bed and used as a CO_2_ methanation reagent. Among the other combustible gases, 33% CH_4_ yield was achieved during the one-stage conversion (T = 500 °C), and 40% methane yield occurred on a two-stage setup (T_1_ = 400 °C, T_2_ = 500 °C) with the use of Ni/600FWB catalyst. The authors have found that the produced methane-rich gas meets the requirements of gas turbines and internal combustion engines [183]. Their approach considers an interesting strategy of using the same raw material both as the source of an activated carbon (which serves as a catalyst support) and also the source of hydrogen required for CO_2_ methanation.

A set of Ni-based composites, supported on ACs derived from the biomass of three different plant species—maple, knotweed, and willow [180] was synthesized and investigated. The study has shown how the choice of raw material determines the properties of the resulting biochar. Among all three materials, the maple leaves-derived biochar exhibited the largest SSA and Ni adsorption capability at the same time, reaching the values of 266 m^2^/g and 165 mg of Ni per 1 g of carbon, respectively. Willow leaves and knotweed leaves-based biochars have shown 40 and 7 m^2^/g SSA, as well as 117 and 105 mg of Ni per 1 g of carbon, respectively. Thus, maple leaves appear to be the best source of biochar among the studied biomass variants. A very important feature of all the obtained biochars is a certain content of alkali metals (mainly Mg and Ca), occurring in the collected biomass samples. It is connected to the place of biomass origin, which was a suburban, post-industrial area, and the alkali metals came from the residues of construction sites. This means that these biochars do not require artificial doping, which generates savings of energy and resources, simplifying the synthetic procedure. Alkali metal contents were measured with the MP-AES technique, and their presence was additionally confirmed by XRD and SEM-EDS results.

Cellulose can be utilized as a source of AC suitable for supporting CO_2_ methanation catalysts as well. Svidersky et al. [184] synthesized Fe-, Co-, and mixed metals-based catalysts. First, the authors obtained a biochar by hydrothermal carbonization of cellulose in a steel autoclave at 190 °C for 24 h. The resulting carbonizate was filtered, dried at 105 °C for 24 h, and calcined in a muffle furnace at 400 °C for 1 h. Fe and Co were deposited on the support from the ethanolic-aqueous solution, and then subjected to an elevated temperature (400 °C) in an inert atmosphere for 1 h. Every catalyst obtained this way had a 20 wt. % metal amount. After activation, the materials were tested in CO_2_ hydrogenation. During the catalytic tests, not only does methane appear in the post-reaction mixture, but higher (up to C_5_) hydrocarbons were also detected, and their content was dependent on the applied Co:Fe ratio. Overall, the higher Fe content favored the formation of longer C-C chains. The pure Co-based composite achieved 48% methane yield at 300 °C and 1 MPa. In comparison, the mixed Co-Fe (1:1) catalyst exhibited only 11% CH_4_ yield at analogous reaction conditions, but it achieved 26% yield of C_5_ hydrocarbons. The authors explain such performance by in situ formation of metallic cobalt (active in CO_2_ hydrogenation) or Co-Fe alloy from carbides during the hydrogenation reaction, which cannot happen when applying metal oxide as a support.

### 4.2. Synthesis Methods

Apart from the classic wet impregnation, scientists apply other, more or less sophisticated synthetic methods to obtain catalysts. Gamal et al. synthesized a set of catalysts supported on sugarcane bagasse-sourced AC [185]. It was prepared according to a very simple one-pot strategy, starting with impregnation of dried sugarcane bagasse with nickel nitrate solution, followed by drying and pyrolysis at 500 °C with a heating rate of 20 °C/min. Next, it was cooled down to room temperature under N_2_ flow. The catalysts had different Ni loadings, depending on the concentration of nickel nitrate solution used for impregnation: 0.1, 0.3, 0.5, 0.8, and 1.2 mmol of nickel per 1 g of sugarcane bagasse powder. The best-performing material from this study, with 0.5 mmol/g Ni, achieved a CH_4_ yield of 33% at 400 °C under atmospheric pressure. At the durability test, it was capable of maintaining its full catalytic potential for 12 h. The authors claim that such activity comes from the high porosity of their materials, resulting in a well-developed specific surface area, and also from the high Ni dispersion observed under SEM and TEM microscopes.

Wang et al. synthesized and characterized Ni-based catalysts supported on Ce-doped ACs, obtained via in situ pyrolysis and activation of pine sawdust [186]. They added pine sawdust to cerium nitrate solution with a biomass:CeO_2_ ratio of 6:1. After ultrasonic treatment, stirring the suspension at elevated temperature, and drying, the authors ground the resulting powder with NaHCO_3_ at a 1:3 mass ratio in a mortar. The next synthesis step was a pyrolysis of activated biomass at 600 °C under a nitrogen flow for 1 h. The product of pyrolysis was subsequently mixed with 0.5 M HNO_3_ and stirred for 4 h at the ambient temperature. Then, the resulting sample was dried at 80 °C overnight to give Ce-doped AC, which was covered with nickel using a modified wet impregnation procedure. The previously prepared support was added to the nickel nitrate solution in ethanol, resulting in a suspension that was ultrasonically dispersed for 20 min, and stirred overnight at ambient temperature. The last step was calcination at 500 °C for 4 h. The synthesis led to obtaining a Ni/Ce-ABC catalyst with a theoretical Ni loading of 15 wt. %. The authors made an interesting assumption that syngas and biochar were the two main products of pine sawdust pyrolysis, and the syngas produced during the process was estimated to provide the amount of heat required for the biochar-based process [187]. Such material was tested in CO_2_ methanation at 200–460 °C, 1 MPa, and a 4:1 H_2_ to CO_2_ molar ratio. As a result, the Ni/Ce-ABC catalyst exhibited 82% methane yield at 360 °C. Moreover, at lower temperatures, the cerium-modified AC-supported catalyst provided even better catalytic performance than the pure CeO_2_-deposited nickel. Cerium improved the stability and dispersion of Ni particles, increasing CO_2_ adsorption at the same time. According to LCA analysis, the preparation of biochar-supported catalyst was more environmentally friendly than the synthesis of its CeO_2_-supported counterpart. Moreover, replacing pure metal-based catalysts with a cheap biochar-based system improved the economic efficiency of CO_2_ methanation.

The same authors developed similar catalytic systems, also supported on pine sawdust-derived ACs, but based on Ru instead of Ni [188]. Also, a different dopant was used in this case—pyridinic nitrogen was implemented into the support structure, utilizing urea as a precursor. The synthetic approach utilized an in situ pyrolysis, combined with nitrogen modification and NaHCO_3_ activation. Various pyrolysis temperatures were used by the authors to produce supports with different physicochemical properties. Mixing pine powder, urea, and sodium bicarbonate at a 1:4:3 mass ratio resulted in obtaining Ru/N-ABC-x (x = 500/600/700) catalysts. According to the results of catalytic tests, the highest methane yield was achieved with the use of the catalyst supported on AC obtained through pyrolysis at 600 °C, meaning that this temperature is optimal for sufficient decomposition of biomass structure and proper porosity development. This catalyst also exhibited the highest content of pyridinic N (38%). The methane yield reached 94% at 380 °C, 1 MPa, and a 4:1 H_2_ to CO_2_ molar ratio. The catalyst was tested at higher temperatures as well, but after reaching the optimum, further increase in temperature resulted in a slight CO_2_ conversion drop due to the exothermic character of CO_2_ methanation. It was proven in another study [189], that if the reaction temperature was increased from 400 to 580 °C, resulting in a change in Gibbs free energy from −40 to 0 kJ/mol, inhibiting the CO_2_ conversion. Another consequence of temperature elevation to such a high level was decreasing CH_4_ selectivity with a simultaneous increase in CO yield due to the occurrence of the methane steam reforming reaction [190].

Li et al. [191] prepared a series of Ni-based catalysts for CO_2_ methanation supported on coconut shell-derived, Mg-doped AC. Composites were synthesized using a co-impregnation method and exhibited better catalytic performance than Al_2_O_3_- and ZrO-supported catalysts. The authors used a fixed-bed tubular flow reactor for catalytic tests conducted under atmospheric pressure and a 4:1 H_2_ to CO_2_ molar ratio. At 450 °C, the catalyst exhibited 89% methane yield. The Ni-Mg_0.26_/coconut shell carbon composite was subjected to a 50 h stability test, and showed no decrease in catalytic performance throughout the entire reaction course.

### 4.3. Doping Elements

The properties of an active phase can be significantly improved by utilizing the doping agents. A highly interesting, Ce-doped material was produced by Mateus et al. [192] from cork wastes. These wastes were physically activated under N_2_-H_2_O (7:3 ratio) atmosphere at 800 °C for 1 h, with 10 °C/min heating rate, reaching 10% yield. The procedure was optimized according to another study made by Mestre et al. [193]. Ni catalyst was prepared by incipient wetness impregnation, and the Ni-CeO_2_ catalyst was synthesized with the use of the co-impregnation method, utilizing respective nitrates as precursors, and isopropanol as a solvent. Both metals’ loadings were established at 15 wt. %, considering the authors’ previous experience with zeolite-supported catalysts, also applied for CO_2_ methanation [194,195]. Next, the samples were dried at 80 °C and heated at 500 °C in a hydrogen flow for 1 h for the purpose of precursor decomposition and obtaining nickel at zero oxidation state. The resulting cork-derived AC-supported catalysts exhibited better affinity toward CO_2_ than composites supported on commercially available ACs. Incorporating CeO_2_ as a dopant for cork-derived AC-supported systems slightly decreased hydrophobicity and caused a further improvement in CO_2_ affinity. The metallic dispersion was also enhanced, resulting in better catalytic performance—the NiCe/AC_N_ catalyst exhibited 69% methane yield at 360 °C.

Tarifa et al. [196] developed a group of Mg-, and/or Ce- doped Ni-based catalysts for CO_2_ methanation, supported on cellulose-derived ACs. The materials were prepared by the incipient wetness impregnation, utilizing nitrates as metal precursors. Ni loading reached 3.5% respectively to unprocessed cellulose. Mg and Ce were applied at various ratios. Samples were thermally decomposed at 600 °C for 3 h under a reducing atmosphere, at a fixed heating rate. The cooldown was performed in an inert gas, followed by passivation with a CO_2_/N_2_ mixture for 1 h. The highest methane yield (80%) was observed in the case of Ni-Mg-Ce/CDC catalyst, at 370 °C and 1 MPa. The authors stated that such performance is a result of the Ce-Mg synergetic doping effect. The beneficial influence of Ce stems from the right combination of Ni reducibility, proper concentration, and basic sites’ strength, uniform size of metal particles, and intensive interactions between Ni and its promoters.

### 4.4. Support Preparation

Support preparation method is another key factor determining the properties of the catalyst. Coconut shell-sourced AC was chosen as a CO_2_ methanation catalyst support also by Le et al. [197]. The authors made a 7 wt. % Ni catalyst supported on biochar doped with ZrO_2_ and CeO_2_ solid solution. At first, the mixed Ce-Zr oxide was obtained following the hydrothermal method scheme, designed by Pham et al. [88]. Cerium nitrate, zirconium chloride, and urea were added to distilled water and stirred to give a complete solution, which was subsequently heated at 160 °C in an autoclave. An obtained product was washed with distilled water until pH stabilization, dried at 80 °C, and calcined at 500 °C for 4 h. The resulting Ce-Zr oxide was deposited on AC using the suspension method: the carbon was mixed with Ce-Zr aqueous slurry, molten surfactant (70 °C, Brij 56), and nitric acid, followed by drying. The as-described procedure was repeated 4 times, and then calcinated at 200 °C for 4 h. The resulting hybrid support was designated as Ce_0.2_Zr_0.8_O_2_/AC. Next, a wet impregnation method was utilized for Ni deposition on the support, which was immersed in nickel nitrate aqueous solution and then dried. The immersing-drying scheme was repeated until running out of nickel nitrate solution. The last step of synthesis was heating at 3 °C/min. rate to 200 °C, and holding this temperature for 4 h. The resulting catalyst was named 7 Ni/CeZrAC. Ce and Zr oxides dispersed and stabilized nickel particles and strengthened the interaction between this metal and the carbonic support. Moreover, these dopants improved Ni reducibility. This resulted in reaching 85% methane yield at 350 °C and atmospheric pressure. The authors claim that these results stem from the interaction between Ni and the doping phase, as well as from the high CO_2_ adsorption capabilities, which come from synergy between three phases: Ni, mixed Ce-Zr oxide phase, and the AC.

The set of Ni/AC composites was synthesized using goldenrod leaves as a carbon source [198]. The results of the study have shown how the pyrolysis time, temperature, pre- and post-treatment of biochar affect the specific surface area of resulting biochars obtained from dried goldenrod leaves. The highest SSA (4 m^2^/g) was achieved on the biochar obtained at 700 °C in nitrogen flow, heating ramp 10 °C/min, held at maximum temperature for 1 h. This biochar was labeled as “2”. Biochars obtained at 600 and 800 °C exhibited smaller SSA, pointing out that 700 °C is an optimal temperature for goldenrod leaves pyrolysis. However, the highest Ni impregnation capacity was not achieved by biochar no. 2, but the “2p” (“p” stands for “powder”, as in the case of this biochar, the dried goldenrod leaves were ground before pyrolysis). Moreover, the SEM-EDS analysis presented an excellent Ni dispersion across the 2p’s surface. The authors claim that the fragmentation of biomass before pyrolysis allows for the uncovering of a larger number of specific functional groups during the heat treatment, resulting in better metal ion adsorption at the biochar’s surface.

## 5. Comparative Assessment of CO_2_ Methanation Catalysts Supported on Biomass-Derived ACs

To evaluate catalysts for CO_2_ methanation, certain criteria must be considered. The first aspect is the catalyst’s performance. It is evaluated by CH_4_ yield, as it is the most important product of CO_2_ methanation. Another key feature is the catalytic system’s durability: the catalysts with higher thermal stability are less prone to deactivation during long-time synthesis and rarely need to be replaced. This means additional savings in resources, energy, waste, and money. Highly durable catalysts are especially valued in industrial synthesis, as large-scale processing systems require a larger catalyst charge per working cycle, and their replacement is often financially unacceptable. The level of the catalyst’s synthesis complexity matters as well, as the fewer steps the catalyst production procedure consists of, the fewer solvents and other chemical agents need to be used during the entire process, including lower energy consumption. The costs of raw materials and their processing are important, as they influence the catalysts’ economic balance. Ecology—for this matter, the material’s renewability and carbon footprint must be considered. The possibility of catalyst recycling raises not only ecological, but also economic value, as material recovery means less waste and no need to consume additional resources.

According to Table 1, among the best-performing catalysts for CO_2_ methanation, three types of systems are exceptionally promising. The first type consists of Ni or Ru particles deposited on cerium- or nitrogen-promoted, pinewood-derived ACs (Ni/Ce-ABC and Ru/N-ABC-600). Such a setup features excellent durability (96% starting catalytic activity after 120 h of methanation in case of Ni/Ce-ABC composite), which makes it perfect for long-term synthesis due to enhanced oxygen mobility, effectively preventing coke deposits formation. The best catalytic performance is exhibited by the Ru/N-ABC-600 catalyst—94% methane yield, definitely outclassing most of the other materials presented in this work. Moderate optimal operating conditions (380 °C, 1 MPa) are also a noteworthy detail, as it provides energy savings. However, the main disadvantage of these catalysts is their synthesis complexity (10 steps), which raises the overall production costs, and in the case of the Ru-based system, the price of the precursor also seems to be repulsive. Multistep synthesis means generating a high amount of waste and extensive energy consumption, but these drawbacks are, at some point, mitigated due to these catalysts’ sturdy structure and impressive thermal stability, making them long-lasting.

The second type of catalysts are Ni-based composites supported on coconut shell-derived ACs (Ni-Mg_0.26_/coconut shell carbon and 7%Ni/Ce_0.2_Zr_0.8_O_2_/AC). Their high methane yield (85–89%) is a very attractive feature, which, combined with the outstanding durability, make them another promising candidate for industrial catalysis. Compared to pine sawdust-derived catalysts, their synthesis is much simpler (3–4 steps), and also the cost of materials is more reasonable by replacing the expensive Ru with Ni. In addition, the 7%Ni/Ce_0.2_Zr_0.8_O_2_/AC shows its best performance at a relatively low temperature (350 °C), requiring less intensive heating and lower energy consumption during methane production.

The third very well-performing and interesting catalytic system is Ni-Mg-Ce/CDC—a Ni-based, Mg and Ce-doped catalyst, supported on cellulose-derived AC. Providing 80% methane yield at 370 °C and 1 MPa, being very durable and relatively simple in production, this material perfectly fits into the idea of a sustainable and eco-friendly catalytic system for CO_2_ methanation. Its synthetic route takes only 5 stages, thus it requires a reasonable amount of energy and solvents, generating a low amount of waste at the same time.

NiCe/AC_N_ is another noteworthy material. It uses Ce- and N- doped AC produced from industrial cork wastes as a support, and Ni as the catalytic active phase. It shows 70% methane yield, while being synthesized from cheap precursors (Ce and Ni nitrates, urea). It was produced in a 5-step synthesis, which makes this material eco-friendly—its preparation does not consume a high amount of energy; also, a large amount of solvents is not necessary. It exhibits an incredible durability as well—during a 24 h catalytic test, it lost only 7% of its initial activity. Moreover, it reaches its peak performance at mild conditions (360 °C, 1 MPa), which helps to save an additional amount of energy for reactor heating and sealing.

An equally interesting composite is a Ni-based, Mn-doped catalyst supported on rice husk-derived, partially oxidized AC (Ni/SiO_2_@C-ox). Its uniqueness lies in applying manganese as a dopant. This relatively cheap transition metal, in interaction with Ni, allows obtaining 68% methane yield at low operating temperature and pressure (350 °C, 1 MPa). Furthermore, the Mn-doped catalyst maintained 100% of its initial activity throughout the whole 24 h durability test. To sum up, Ni/SiO_2_@C-ox is a low-cost catalyst with a decent performance and excellent durability, which can operate at mild conditions. However, the drawback of this material is its synthesis complexity—it consists of eight stages, meaning a significant energy and solvent consumption, along with chemical waste generation. This feature does not disqualify the catalyst in terms of industrial application, but it downgrades its value at a certain point.

Wheat straw-derived AC-supported catalysts, the BCNiCe30 and BCCe30Ni40, are Ni-based, Ce-supported catalysts that show an average catalytic performance (54 and 60% CH_4_ yield, respectively). Their durability is rather mediocre—the BCNiCe30 had lost most of its starting activity after 14 h of non-stop catalysis at its optimal working conditions. This significant drawback makes this catalyst inapplicable in industrial methane synthesis through CO_2_ reduction. A specific feature of this system is that it prefers relatively high operating temperatures—the optimal value for this particular catalyst is 450 °C—but on the other hand, it can work at very low pressure—only 0.1 MPa. Its synthesis route has a standard complexity (five stages), and the precursor costs are average. BCCe30Ni40’s durability is unknown at this moment, It operates at a milder temperature (350 °C) than BCNiCe30, but at 10 times higher pressure (1 MPa). Also, its preparation process is more complicated—7 stages instead of 5—which means higher production costs and greater resource consumption.

A set of parameters opposite to that of pine sawdust-derived AC-supported catalysts can be observed for reed-, cellulose-, and sugarcane bagasse-derived AC-supported catalysts. Their production costs are very low because they utilize Ni as the active phase; their supports are based on waste and highly abundant, low-value materials; no dopants are incorporated into their structures; and their synthetic procedures are straightforward. Unfortunately, the catalytic performance of these materials is very low, and because of this feature, their industrial application is strongly questionable.

To begin with, the catalyst supported on cellulose-derived AC (Co/biochar) shows a mediocre yield of methane(only 48%). Also, its long-term stability remains unknown. In comparison with a similar system, the Ni-Mg-Ce/CDC, a clear conclusion can be made: Co is not the right element to serve as an active phase of a catalyst dedicated to CO_2_ methanation. This comparison shows the meaning of dopant elements as well—Mg and Ce used as promoters greatly enhanced Ni deposits’ catalytic performance. The most important assets of Co/biochar catalyst are its low production cost (no dopants, no significant modifications made to the support, relatively cheap active phase metal precursor), and low operating temperature (300 °C). However, they are not significant enough to balance the catalyst’s poor performance.

The activity of the reed-derived AC-supported, Ni-based catalyst (Ni/FWB-600) is even worse. This catalyst presents a low methane yield (40%), Its optimal operating pressure does not seem optimistic as well—it reaches a high 500 °C value, resulting in extensive energy consumption during methane synthesis. These parameters disqualify this catalytic system in terms of industrial application. The only positive side of this material is a low preparation cost, which stems from the absence of support modifications, applying Ni as the active phase, no activation done to the biochar, and the use of a cheap and abundant carbon precursor.

0.3Ni/SCBB—this sugarcane bagasse-derived, AC-supported, Ni-based catalyst stands as a total opposite to the Ru/N-ABC-600 catalyst. 0.3Ni/SCBB’s catalytic performance is exceptionally low, as it exhibits 33% methane yield. It operates at a moderate temperature of 400 °C and low pressure—1 MPa. Its synthetic procedure is simple, consisting only of four steps. Combined with the low price of both support and active phase precursors, this catalyst appears to be the cheapest among all presented in this review. Moreover, the 0.3Ni/SCBB is surprisingly durable—it maintained its full activity during the whole 12 h CO_2_ methanation test. Considering these assets, this material also seems to be the most eco-friendly of all the catalysts described above.

All in all, Ni-Mg_0.26_/coconut shell carbon seems to be the best catalyst among all compared in this work. Its excellent performance-to-cost ratio, as well as its eco-friendly character due to high durability and three-step synthesis, make this material highly applicable for a large-scale, industrial CO_2_ methanation.

## 6. Conclusions

To conclude, nickel-based catalysts supported on biomass-derived activated carbons are a very promising class of materials for CO_2_ methanation. Their advantages include mostly low-cost, tunable properties, and compatibility with renewable feedstocks. Currently, an increasing focus on waste biomass utilization can be observed. Scientists put a lot of effort into the development of hierarchical pore structures that can be applied as advanced, customizable catalyst supports, allowing greater dispersion of transition metals and better heat distribution during CO_2_ methanation. A very important aspect of modern catalyst development is also the incorporation of promoters into the metal/AC composites to enhance their stability and catalytic activity. The key challenge in catalyst design is the optimization of metal–support interactions, as they are the essential factor influencing the durability of catalysts. High pressure is applied to large-scale AC production from various types of biomass.

## 7. Future Perspectives

Recently, strong attention has been paid to the exploration of hybrid catalysts’ supports, mainly considering carbon + metal oxide-type materials. Also, synergy between catalysts’ promoters is studied, especially considering interactions between alkali earth metals and heteroatoms (mainly N). After optimization of CO_2_ methanation catalysts, the next important step is their incorporation into industrial Power-to-Gas systems, which is going to be their main application in the near future, and the main studied topics are going to be the convergence of catalysis, materials science, and circular economy principles. These prospects reveal biomass-derived Ni/AC catalysts as key contributors to future low-carbon energy systems.

## Figures and Tables

**Table 1:** Comparison of the most important parameters of CO_2_ methanation catalysts supported on biomass-based ACs. In the 8th column, value 1 means the lowest production cost, while value 5 means the highest.

Catalyst Name	Biomass Source	Active Phase	Preparation Stages	CH_4_ Yield [%]	Operating T and *p* [°C, MPa]	Cost (1–5 pts.)	Durability [%, h]
BCNiCe30 [171]	Wheat straw	Ni, Ce	5	60	450, 0.1	2	30,14
BCCe30Ni40 [173]	Wheat straw	Ni, Ce	7	54	350, 1	3	unknown
Ni/SiO_2_@C-ox [134]	Rice husk	Ni, Mn	8	68	350, 1	3	100,24
0.3Ni/SCBB [185]	Sugarcane bagasse	Ni	4	33	400, 1	1	100,12
Ni/Ce-ABC [186]	Pine sawdust	Ni, Ce	10	82	360, 1	4	96, 120
Ru/N-ABC-600 [188]	Pine sawdust	Ru, N	10	94	380, 1	5	unknown
Ni-Mg_0.26_/ coconut shell carbon [191]	Coconut shell	Ni, Mg	3	89	450, 1	2	100, 50
7%Ni/Ce_0.2_Zr_0.8_O_2_/AC [197]	Coconut shell	Ni, Ce, Zr	4	85	350, 1	3	unknown
NiCe/AC_N_ [198]	Cork wastes	Ni, Ce, N	5	70	360, 1	3	93, 24
Ni/600FWB [179]	Reed	Ni	5	40	500, 1	2	unknown
Co/biochar [184]	Cellulose	Co	5	48	300, 1	1	unknown
Ni-Mg-Ce/CDC [196]	Cellulose	Ni, Mg, Ce	5	80	370, 1	3	95, 17

## Data Availability

No new data were created or analyzed in this study. Data sharing is not applicable to this article.

## Abbreviations

The following abbreviations are used in this manuscript:

AC	Activated Carbon
ORR	Oxygen Reduction Reaction
HER	Hydrogen Evolution Reaction
GHSV	Gas Hourly Space Velocity
CCU	Carbon Capture and Utilization
